# The two-fluid dynamics and energetics of the asymmetric magnetic reconnection in laboratory and space plasmas

**DOI:** 10.1038/s41467-018-07680-2

**Published:** 2018-12-06

**Authors:** M. Yamada, L.-J. Chen, J. Yoo, S. Wang, W. Fox, J. Jara-Almonte, H. Ji, W. Daughton, A. Le, J. Burch, B. Giles, M. Hesse, T. Moore, R. Torbert

**Affiliations:** 1 Princeton Plasma Physics Laboratory, Princeton University, Princeton, NJ 08543 USA; 2 NASA Goddard Space Flight Center, Greenbelt, MD 20771 USA; 3 Los Alamos National Laboratory, Los Alamos, NM 87544 USA; 4 Southwest Research Institute, San Antonio, TX 78238 USA; 5 University of Bergen, Bergen, 5007 Norway; 6 University of New Hampshire, Durham, NH 03824 USA

## Abstract

Magnetic reconnection is a fundamental process in magnetized plasma where magnetic energy is converted to plasma energy. Despite huge differences in the physical size of the reconnection layer, remarkably similar characteristics are observed in both laboratory and magnetosphere plasmas. Here we present the comparative study of the dynamics and physical mechanisms governing the energy conversion in the laboratory and space plasma in the context of two-fluid physics, aided by numerical simulations. In strongly asymmetric reconnection layers with negligible guide field, the energy deposition to electrons is found to primarily occur in the electron diffusion region where electrons are demagnetized and diffuse. A large potential well is observed within the reconnection plane and ions are accelerated by the electric field toward the exhaust region. The present comparative study identifies the robust two-fluid mechanism operating in systems over six orders of magnitude in spatial scales and over a wide range of collisionality.

## Abstract (web summary)

Magnetic energy in the plasma is transferred into particle energy by magnetic reconnection. Here the authors show the two-fluid dynamics of asymmetric magnetic reconnection in two different spatial scales of plasma, namely laboratory and astrophysical plasma.

## Introduction

Fast magnetic reconnection, the breaking and reorganization of magnetic field lines, occurs where two regions of magnetized plasma with nearly opposite magnetic field line directions meet. The movement of magnetic field lines caused by external forcing leads to the formation of a current layer wherein magnetic reconnection occurs. The reconnection speed is characterized by the amount of field lines moving from one section of topology to the other. In the past two decades, our understanding of the physics of fast reconnection has significantly improved through numerical simulations, observations from space satellites, and from dedicated laboratory plasma experiments^1–10^. It is now understood that two-fluid effects^1–6^ caused by the decoupling between ions and electrons, play a key role within the reconnection layer, and the Hall effects have been shown to facilitate fast reconnection observed in collisionless magnetospheric plasmas and nearly collision-free laboratory plasmas. As magnetized electrons are moving along the field lines, they also flow in the out-of-reconnection-plane direction being simultaneously accelerated by the reconnection electric field, pulling the field lines in the out-of-plane direction. This pulling deforms the reconnecting field lines to generate the out-of-plane quadrupole field together with fast reconnection^2,8^

With recent launch of Magnetospheric Multiscale Satellite (MMS)^11^, the focus of reconnection research has been turned to the dynamics and energetics of asymmetric reconnection in which the plasma density of the one side of inflow region is significantly larger than the other (by factor of 10 or more). This is one of the most important features of the magnetopause reconnection, in which a pile-up of the solar wind plasma density is significantly larger than the magnetosphere density by a factor of 10–50. In the reconnection layer at the magnetopause, the solar wind plasma pressure balances with the magnetic field pressure of the earth dipole field; *β*~1. In the reconnection layer, the thickness of the current sheet becomes comparable to the ion skin depth as well as the ion gyro-radius^1,2^. Ions become demagnetized within the reconnection region as the magnetic field strength becomes small, while electrons are still magnetized and remain frozen to field lines until they reach very near the X-line. This reconnection regime is often called the two-fluid regime. In the vicinity of X-line, even electrons become demagnetized and diffuse, thus we call this region electron diffusion region.

Recently, a laboratory study on the mechanisms of energy conversion and energy partitioning made significant progress towards understanding these issues in a nearly collision-free environment^12–14^. The simultaneous measurements by a few hundred magnetic probes can capture global features of field evolution in the reconnection layer in the MRX (Magnetic Reconnection Experiment) plasma^2,7,8^. On the other hand, coordinated MMS measurements by four satellites can document detailed local properties including measurements of the velocity space particle distributions. Thanks to the self-similar scaling^15,16^, both MRX and the magnetosphere plasma systems reside in the regime of magnetic reconnection in which two-fluid physics dominates^1,2,8^. It is recognized that key parameters of both systems indicate that the scale size of the reconnection region is about three times the ion skin depth, (*d*_i_ = *c*/*ω*_pi_). This allows an unprecedented level of cross-examination between laboratory measurements and space observations. Moreover, magnetic reconnection in both MRX and the magnetosphere is driven by external forcing, i.e., flux cores in MRX and the solar wind in the magnetosphere. For example, the observational verification of electrons’ motion frozen to field lines outside of the electron diffusion region^11^ matches well the earlier measurements in MRX^12,14^ in which the electron dynamics are analyzed in terms of two-fluid physics in symmetric reconnection^8,12^. The excellent agreement demonstrates that the same two-fluid mechanisms in 2D analysis operate well in both systems despite vastly different scales size (1–2 × 10^6^) and collisional conditions, despite various 3D phenomena including micro-fluctuations are expected to be involved.

The energetics of the symmetric reconnection layer was already studied in the context of two fluid physics in MRX^13,14^. In the reconnection layers without guide field, it was found that the energy deposition to electrons primarily occurs through **j**_e⊥_·**E**_⊥_ (where **E**_⊥_is the electric field perpendicular to the magnetic field and **j**_e_ is the electron current density) and is concentrated in the electron diffusion region where electrons are demagnetized and diffuse.

Here we directly compare the dynamics and energetics of asymmetric, anti-parallel reconnection layer observed both in the laboratory plasma of MRX^7–10^ and in the magnetopause by MMS^11^ and discuss our results in the context of two-fluid physics, aided by numerical simulations. Our experimental analysis both on MRX and MMS demonstrates that the primary energy deposition on electrons occur again through **j**_e⊥_·**E**_⊥_, which is now strong at the stagnation point located near the X-line. The energy deposition to ions is observed to be due to **j**_i⊥_·**E**_⊥_ in the exhaust region^10^, where **j**_i_ is the ion current density. The potential well is observed to shift toward the lower density side of the exhaust region in MRX^10^. As was the case in the symmetric reconnection, the accelerated ions are thermalized by re-magnetization in the downstream region with some additional collisional effects. A quantitative inventory of the converted energy is also documented in an asymmetric reconnection layer in MRX with a well-defined boundary and compared with the results from numerical simulations. While the MMS measurements from limited amount of satellite paths could not allow a quantitative assessment, the MRX and numerical studies concluded that a significant part (more than 50%) of the inflowing magnetic energy is converted to particle energy in remarkable agreement both for symmetric and asymmetric reconnection layers.

## Results

### Laboratory experiment

Recognizing that the scale of both MRX and MMS reconnection region is about three times the ion skin depth (*d*_i_ = *c*/*ω*_pi_), we have carried out systematic comparative study of the dynamics and energy flows of the reconnection layer.

Table 1 shows the plasma parameters of MRX and the magnetopause in order to show that they are in the same two-fluid regime, namely different motions of ions and electrons, with the same normalized scale length ~3. In the both systems, the density asymmetry of the inflowing plasma is about 10. Additionally, the Lundquist number, *S* (the ratio of resistive magnetic diffusion time to the Alfvén transit time) is also significantly larger than 1 (*S* ≫1)^1,2^, which makes it possible to describe global plasma dynamics by ideal MHD except at the reconnection layer. Due to the above self-similar conditions and relationship, reconnection in MRX is expected to share key qualitative and quantitative characteristics with reconnection in the magnetosphere in terms of plasma dynamics and energetics.

**Table 1:** Plasma parameters and scale sizes of MRX and at the magnetopause

	MRX	Magnetopause	Ratio
System scale size (*L*) Half-length of reconnection layer	0.1–0.2 m	100–200 km	~10^6^
Ion skin depth (*d*_i_ = *c*/*ω*_pi_)	4–8 cm	40–80 km	~10^6^
Electron skin depth (*d*_e_ = *c*/*ω*_pe_)	0.5–2 mm	1–2 km	~10^6^
Normalized scale length (L^* = ^*L*/*d*_i_)	2–4	2–4	~1
Density ratio across the current sheet	5–30	10–100	~1
Plasma beta on the high-density side (*β*_high_)	0.5–1	1–4	~1
Plasma beta on the low-density side (*β*_low_)	0.05–0.3	0.1–0.6	~1
Lundquist number (*S*)	≳10^3^	>10^10^	~10^7^

The ion and electron skin depths are based on the density on the high-density (magnetosheath) side. The plasma beta is the ratio of the plasma pressure (*p*) to the local magnetic pressure; *β* = *p* /(*B*^2^/2*μ*_0_). Note that *S*»1 is satisfied for ideal MHD to be valid globally in both cases

**Fig. 1:**
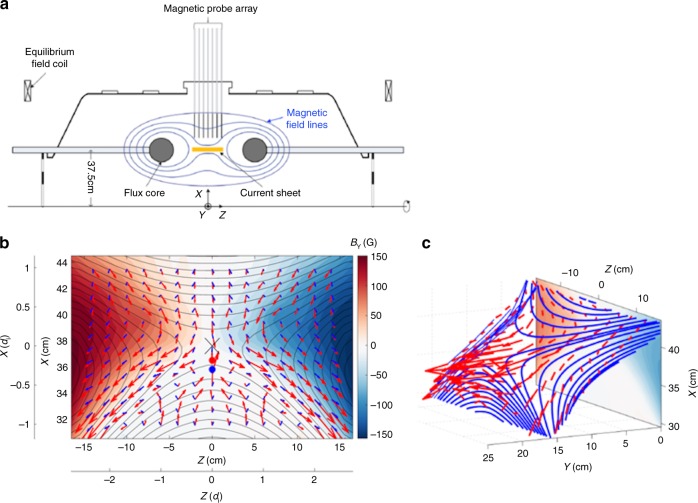
MRX apparatus and demonstration of the two fluid effects. **a** MRX apparatus to generate asymmetric reconnection the current sheet. Each flux core (gray circles) contains two sets of internal coils that are used to create plasma and to drive reconnection^7^. The distance between the surfaces of the two flux cores is 40 cm. By controlling the sequence of coil currents and the initial plasma flows, asymmetric reconnection is formed with electron density asymmetry^9,10^ of up to 10. **b** Measured flow vectors of electrons (red arrows) and ions (blue arrows) in the full reconnection plane together with poloidal flux contours (black lines), and color contours of the out-of-plane magnetic field. The marker *X* at (*X*, *Z*) = (37.6, 0) cm denotes the location of the *X*-line where magnetic field is near zero, the red filled circle at (*X*, *Z*) = (36.5, 0) cm is the stagnation point of in plane electron flows, and the blue circle at (*X*, *Z*) = (35.8, 0) cm is the stagnation point of ion flows. The separate axes are provided to indicate the size of the measurement region in the ion skin depth (*d*_i_). **c** 3D view of reconnecting magnetic field lines. The movement of the field lines in the reconnection plane can be tracked in Supplementary Movie 1 (attached in this paper) from MRX data. Because of the dipole structure of out-of-plane magnetic field due to the Hall effect, the plane where field lines move with electrons is tilted with respect to the *Y*-axis on the high-density side

The MRX facility is used to experimentally study asymmetric reconnection. Figure 1 shows a schematic of MRX (Fig. 1a) together with the measured flow of electrons and ions in the reconnection layer overlaid on contours of the poloidal flux (Fig. 1b). Experiments are carried out in a setup in which two toroidal plasmas, each with an annular cross section, are formed around two flux cores (gray circles in Fig. 1a). Each flux core contains both toroidal field (TF) and poloidal field (PF) coils. By controlling the currents in the two coils, we can routinely generate the reconnection layer in a controlled manner and detailed plasma parameters are measured by internal probes^7,8^. By controlling the sequence of coil currents and the initial plasma flows, asymmetric reconnection is formed with electron density asymmetry^9,10^ of a factor 8–10.

For standard conditions, *n*_e1_ (high-density side) ~4 × 10^13^ cm^**-**3^, *n*_e2_ (low-density side) ~5 × 10^12^ cm^**-**3^, *T*_e_ = 5–15 eV, *B* = 0.1–0.2 kG, *S* (Lundquist number) >500; the electrons are mostly magnetized (gyro-radius, < *ρ*_*e*_ > ~c/*ω*_pe_ ~ 1 mm « *L*, where *L* is the length of the reconnection layer), while the ions are not. The mean free path for electron-ion Coulomb collisions is in the range of 5–30 cm (>the layer thickness), and, as a result, the reconnection dynamics are dominated by two-fluid and kinetic effects^2,8^, despite some collisional effects were seen^9^. In our coordinate system (*X, Y, Z*), *B*_*Z*_ is the reconnecting field component and *Y* is along the out-of-plane axis.

Using ion dynamics spectroscopy probes (IDSPs) to measure the ion temperature^12^, about 300 pieces of magnetic probes to measure the vector magnetic field **B**, Mach probes to measure ion flows^12^, and triple Langmuir probes to measure electron temperature and density, we have studied the comprehensive dynamics of the plasma within the reconnection layer and without an externally imposed guide field. Figure 1b depicts flow vectors of ions (in blue) and electrons (red) across the whole reconnection plane, along with poloidal flux contours and colored contours of the out-of-plane magnetic field component, *B*_*Y*_. It should be noted that the high-density side of the reconnection plane in which magnetic field lines move with electrons, is strongly tilted with respect to the *y*-axis, as shown in Fig.1c. This unique feature of the asymmetric reconnection is caused by decoupling of electrons and ions, Hall effects which generate a dipole field shown in Fig. 1b^10^. The Hall current is carried by electrons flowing toward (away from) the X-line in the high (low)-density side as seen in Fig. 1b. The electron flows toward the X-line in the separatrix region of the high-density side have been also measured by MMS at the magnetopause in agreement with Fig. 1b^17^. This measurement was verified in the 2D simulation as described in Methods section.

The electron flow vectors in the reconnection plane are derived from the electron current profile, which is obtained from the magnetic field profile measured by fine scale magnetic probes. Specifically, we use the equations **J** = ∇×**B** /*μ*_0_ and **U**_e_ **=** −**J/***n*_e_*e* *+* **U**_i_ to compute the electron flow: **U**_e_ and **U**_i_ denotes flow velocity of electrons and ions, respectively. The measured flow profiles of electrons and ions clearly demonstrate that two-fluid reconnection is at work in MRX. Ions, which become demagnetized as they enter the ion diffusion region, whose width is *d*_i_ = *c*/*ω*_pi_ (5–6 cm), and are accelerated across the separatrices, flowing outward to the exhaust direction, as seen in Fig. 1b. In contrast to the case of symmetric reconnection, we observe that inflowing ions also form a stagnation point (denoted by a blue circle) near the X-line (X-point) on the low-density side with a shift of about 2–3 cm (0.3–0.5*d*_i_). This was also verified in the 2D simulation as described later.

### Energy deposition and structure of the electron diffusion region

One of the most important results of MRX-MMS collaboration has been to clarify the role of the electron diffusion region together with the energy deposition to electrons. In both measurements, we identified a two-scale diffusion layer in which an electron diffusion layer (half width δ~ 5 mm in MRX; 5–10 km in MMS) resides inside of the ion diffusion layer (half width *δ*_i_ ~ 6 cm in MRX (He gas) and ~100 km in MMS), the half width of which is about 100 times the electron skin depth^2,11^. In this situation, the ion diffusion layer is defined by the regime where **E** + **U**_i_ × **B** ≠0 with **E** + **U**_e_ × **B** ≈ 0 and. The electron diffusion layer is the regime of **E** + **U**_e_ × **B** = **E**^**′**^ ≠0, where **E**^**′**^ is the electric field in the electron fluid frame. Just outside the electron diffusion layer, **E**^**′**^ = 0 holds, namely electrons move with magnetic field lines in the reconnection plane (electron-flux freezing), and this relationship was clearly verified by Burch et al.^11^ and by quantitatively evaluating force balance in MRX^12^. We note that in the case of MRX, the difference between **E** and **E**^**′**^ is relatively small since *U*_e_ is much smaller than the electron thermal velocity, *U*_e_ ~ 0.1*V*_eth_, where *V*_eth_ is the electron thermal velocity, but this does not apply for MMS data.

**Fig. 2:**
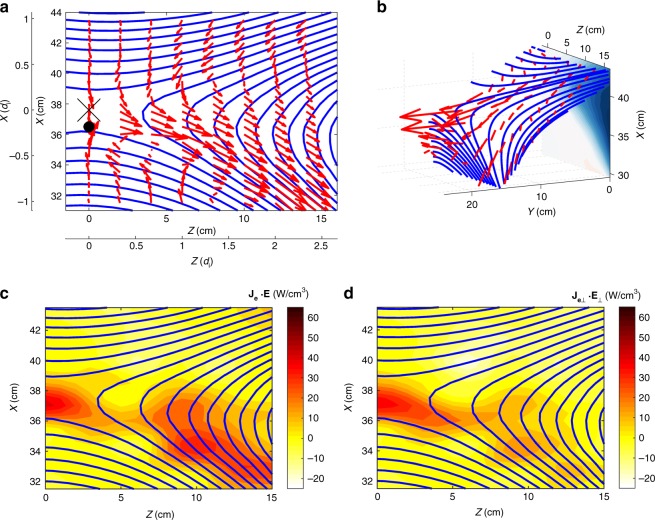
Electron dynamics observed in MRX. **a** In the reconnection plane, electron flows together with reconnecting field lines. The X marker at (*R*, *Z*) = (37.6, 0) is the X-line and the black circle denotes the stagnation point of in-plane electron flow. **b** 3D views of electron flow vectors with respect to the reconnecting field lines. Because of the dipole structure of out-of-plane magnetic field, the reconnection plane is tilted in the *Y*-axis. in the high density (upper) side. Note that strong electron current in *Y*-direction at the stagnation point. **c** Energy deposition to electrons through **j**_e_·**E** is concentrated in the electron diffusion region around the stagnation point as well as in the lower density side of exhaust. **d** Perpendicular component, **j**_e⊥_∙**E**_⊥_ is much larger than *j*_e||_*E*_||_ and concentrated in the electron stagnation point, while the contribution from *j*_e||_*E*_||_ is notable at the lower density side of the exhaust as seen in **c** and **d**

In the asymmetric MRX experiments, we observe distinctly different flow patterns as compared to the symmetric case which was reported previously^13^. Figure 2a, b present measurements of the electron flows in 2D and 3D views within one half of the reconnection plane. As seen in Fig. 2a, b, electrons are flowing out in the *Y* direction as well as toward the exhaust in the *Z* direction. The out-of-plane electron drift velocity becomes very large at the stagnation point of in-plane electron flows, which is located near the X-line but shifted toward the lower density side by several electron skin depths (0.5–1.0 cm ~5–10(*c*/*ω*_pe_)). The out-of-plane magnetic field nominally exhibits a quadrupolar pattern during symmetric reconnection, a signature of Hall effect, but is modified significantly during asymmetric reconnection due to shifted patterns of Hall currents as seen in the color contours of Fig. 1b. Due to this nearly bipolar structure seen in Fig. 1b, the reconnection plane, wherein reconnecting field lines move in together with electrons, is tilted to the *Y* direction as shown in Fig. 2c. It should be noted that the tilt is strong in the high-density inflow region due to the stronger out of plane Hall field component. The electrons which move together with magnetic field lines flow in the tilted plane in the high-density side, become demagnetized in the electron diffusion region, and stream out in the *Y* direction as well as in the *Z* direction.

When the energy deposition rate to electrons, **j**_*e*_·**E**, is decomposed into **j**_e⊥_∙**E**_⊥_ + *j*_e*||*_*E*_||_, i.e. separating the inner product into that of the perpendicular and parallel components with respect to the local magnetic field lines, **j**_e⊥_∙**E**_⊥_ is measured to be significantly larger than *j*_e*||*_*E*_||_ near the X-line. Near the electron stagnation point, **j**_e⊥_∙**E**_⊥_ is larger than *j*_e*||*_*E*_||_ by more than an order of magnitude.

**Fig. 3:**
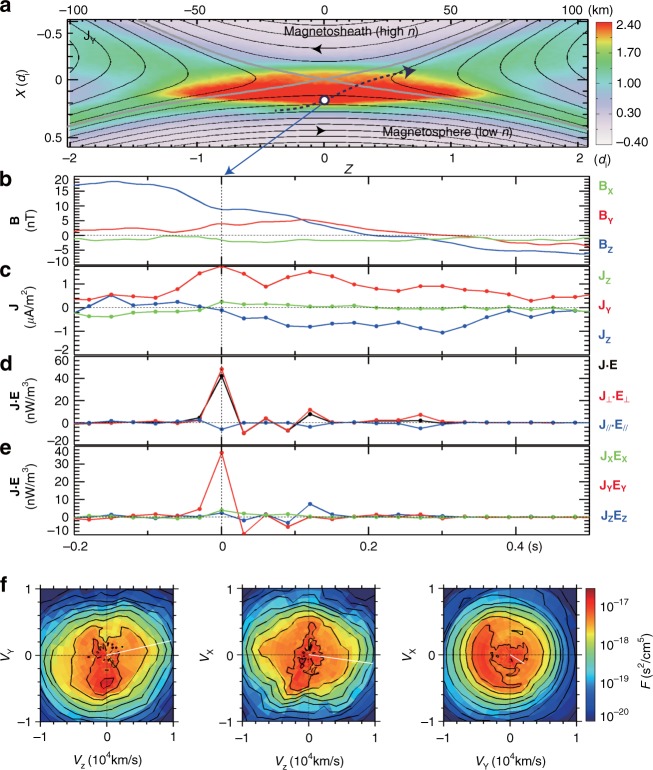
MMS measurements of the electron diffusion region with negligible guide field. Energy deposition to electrons peaks at the electron diffusion region. **a** Path of MMS through the electron diffusion region illustrated on the out-of-plane current density (*J*_*Y*_) contours from a 2D PIC simulation. **b** The magnetic field components. **c** The components of the current density. **d** The value of **J**∙**E** ~ **J**_⊥_∙**E**_⊥_~ **j**_e⊥_∙**E**_⊥_ spikes up when the spacecraft MMS2 encounters the electron diffusion region. The encounter at the center of the electron diffusion region occurs around the time of *t* = 0. **e** The **J**∙**E** is dominantly contributed by *J*_*Y*_*E*_*Y*_ *~ j*_e*Y*_*E*_*Y*_. **f** Electron velocity distribution function in three orthogonal velocity planes. The distribution is from the peak **J**∙**E** (time marked by a vertical dotted line in **b**–**e**; motion of the MMS satellites is marked by a black open circle in **a**). The data are plotted in the frame of the X-line. The direction of the electron flow vector from the velocity distributions shown in **f** is consistent with that of the electron flow vector measured in MRX (Figs. 1c, 2b)

It was demonstrated in MRX^13,14^ that in symmetric reconnection without guide field, the energy dissipation to electrons occurs primarily due to **j**_e⊥_∙**E**_⊥_ only near the center of the electron diffusion region, the X-line. However, in asymmetric reconnection, it is verified that **j**_e⊥_∙**E**_⊥_ peaks up through *j*_ey_*E*_y_ at the stagnation point of the electrons’ in-plane flow, which is separated from the X-line by ~ 5–8 *c*/*ω*_pe_. Recent analysis of data from MMS also verified this key feature by demonstrating that the value of **j**_e⊥_∙**E**_⊥_ peaks when MMS encounters the electron stagnation point in the electron diffusion region as will be discussed in Fig. 3 in the next section.

### MMS observation of the electron diffusion region

To investigate where and how energy conversion occurs in magnetopause reconnection, we discuss an encounter with both the electron and ion diffusion regions by the MMS spacecraft on 16 October 2015. The event has a negligible guide field, and the general features are described in Burch et al.^11^ The plasma density and flow velocity, magnetic field, and electric field measurements shown in Fig. 3 are from the Fast Plasma Investigation^18^, the FluxGate magnetometers^19^, and the double-probe electric field sensors^20^, respectively.

To facilitate the comparison with MRX experimental results, we here use the same coordinate system (*X, Y, Z*) which corresponds to (*N, -M, L*) in the boundary normal *LMN* coordinates determined in Denton et al.^21^. We note that this (*X*, *Y*, *Z*) coordinate differs from the Geocentric Solar Magnetospheric (GSM) system.

Figure 3 presents energy conversion to electrons in the electron diffusion region during magnetopause reconnection. The kinetic structure of the same diffusion region, including higher frequency fluctuations, has been previously discussed^11,21^. Here we filter out electric field fluctuations (such as whistler and lower-hybrid waves) with frequencies higher than the ion cyclotron frequency in order to observe the longer-time-scale structure of the plasma dynamics and energy conversion.

Figure 3a shows the approximate MMS trajectory through the electron diffusion region on the profile of *J*_*Y*_. The trajectory is determined based on our comparative study of the MMS measurements and PIC predictions of electron distribution functions and reversals of *E*_*X*_, *B*_*Z*_, *U*_iz_, *U*_e*z*_, and *J*_*Y*_^22^. The average velocity of the magnetopause plasma (X-line included) along the *X* axis during the electron diffusion region crossing is about −30 km s^−1^ based on the four-spacecraft magnetic field measurements, and the velocity along *Z* is estimated to be −97 km s^−1^ ^21^.

Energy deposition to electrons peaks at the electron diffusion region through the work done by the reconnection electric field as demonstrated in Fig. 3. The amplitude of the out-of-plane current density *J*_Y_ (Fig. 3c) is enhanced when MMS encounters the electron diffusion region and particularly the vicinity of the electron stagnation point where *J*_*X*_ and *J*_*Z*_ reverse signs (labeled as *t* = 0 in Fig. 3b–e). Note that in the frame of the reconnection X-line, **J** is dominated by the electron current density **j**_**e**_. The energy conversion **J**∙**E** is primarily contributed by **J**_⊥_∙**E**_⊥_***~*** **j**_e⊥_∙**E**_⊥_ (Fig. 3d) when decomposed into parallel and perpendicular, and by *J*_*Y*_*E*_*Y*_ *~ j*_e*Y*_*E*_*Y*_ when decomposed into the *X*, *Y*, *Z* terms (Fig. 3e). In essence, the energy deposition rate to electrons is the highest around the electron stagnation point in the electron diffusion region, and as demonstrated in Fig. 3e, the energy deposition is through the work done by the reconnection electric field *E*_*Y*_. *E*_*Y*_ is about 1.6 mV m^−1^ near the X-line. This value translates to a reconnection inflow velocity of 0.23 V*_A_, where V*_A_ is the hybrid Alfvén velocity^23^. This measured value of the reconnection electric field agrees with the MRX data, which shows 140 V m^−1^ of *E*_*Y*_ that corresponds to an inflow velocity of 0.25 V*_A_ (*B*_1_ = 175 G and *B*_2_ = 200 G; *B*_1_ and *B*_2_ are the strength of the reconnecting magnetic field component on the high- and low-density sides, respectively).

In the peak energy-conversion region defined by the high **j**_e⊥_∙**E**_⊥_, a crescent shaped electron distribution function was detected, showing a strong electron flow along *Y*, the out-of-reconnection-plane direction (Fig. 3f). The distribution function provides the kinetic view for the strong electron out-of-plane flow along *Y* in MRX, as seen in Fig. 2b.

In summary, the above observations from MMS in Fig. 3 show an excellent agreement with the MRX results in which electrons flow out of the stagnation point just underneath the X-line towards *Y* and *Z* directions as demonstrated in Fig. 2b making significant contribution to energy conversion through **j**_e_·**E** as indicated in Fig. 2d.

### Ion dynamics and energetics in MRX and the magnetosphere

**Fig. 4:**
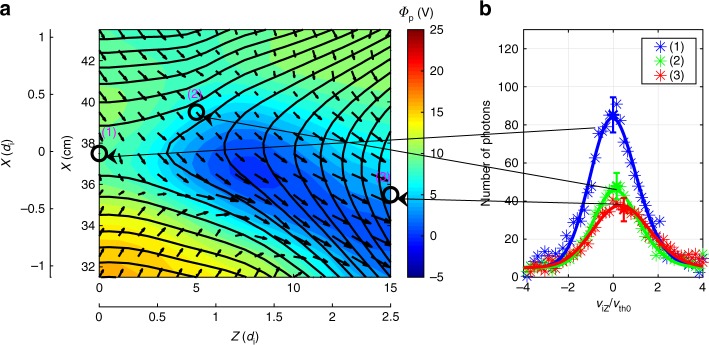
Electrostatic potential profile and ion acceleration during asymmetric reconnection in MRX. **a** The well of the saddle back shaped potential is shifted toward the low-density side and a sharp potential drop occurs in the low-density side of the exhaust region. **b** The ion velocity distribution in *Z* direction was measured by the IDSP spectroscopically. The peak of the ion distribution function in *V*_iz_ increases to higher values toward downstream. Measured velocity *V*_iz_ is normalized by the ion thermal velocity at the X-line. Compared to the symmetric case^13^, ions are not significantly accelerated near the separatrix on the high-density side (green line). The ion temperature in the exhaust region (red) is also slightly lower. The change of the in-plane potential profile is responsible for these differences. Error bars are the square root of the number of photons

The large in-plane electric field plays a key role in ion acceleration and heating^10,12^. The recent studies identified the mechanisms how the in-plane electrostatic field is generated by the force balance of the electrons flowing through the center of the reconnection layer. During symmetric reconnection in MRX, it was found that the measured in-plane electric potential profile is of a saddle back shape and the resulting electric field is three times larger than the reconnection electric field^12–14^. However, during asymmetric reconnection, the saddle back shaped potential well is shifted toward the low-density side and a sharp potential drop occurs on the low-density side of the exhaust region as shown in Fig. 4a^10^. The unmagnetized ions are accelerated by the in-plane electric field in the exhaust region both in *Z* and *X* directions primarily in the high-density side and are heated further downstream. This is in contrast to the symmetric case in which ions are accelerated on both sides of the separatrices. For the case of symmetric reconnection, the value of **j**_i⊥_·**E**_⊥_, which is the ion energy gain per unit time and unit volume is about 30–40 W cm^−3^ near the separatrices^12–14^. For the asymmetric case is reduced to about 15 Wcm^−3^, although the ion acceleration region is notably wider. Figure 4b presents velocity space distribution of ions at three locations denoted in Fig. 4a. One can see that the ions are drifting down stream with an elevated temperature which is caused by stochastic motions of ions and some collisional effects.

**Fig. 5:**
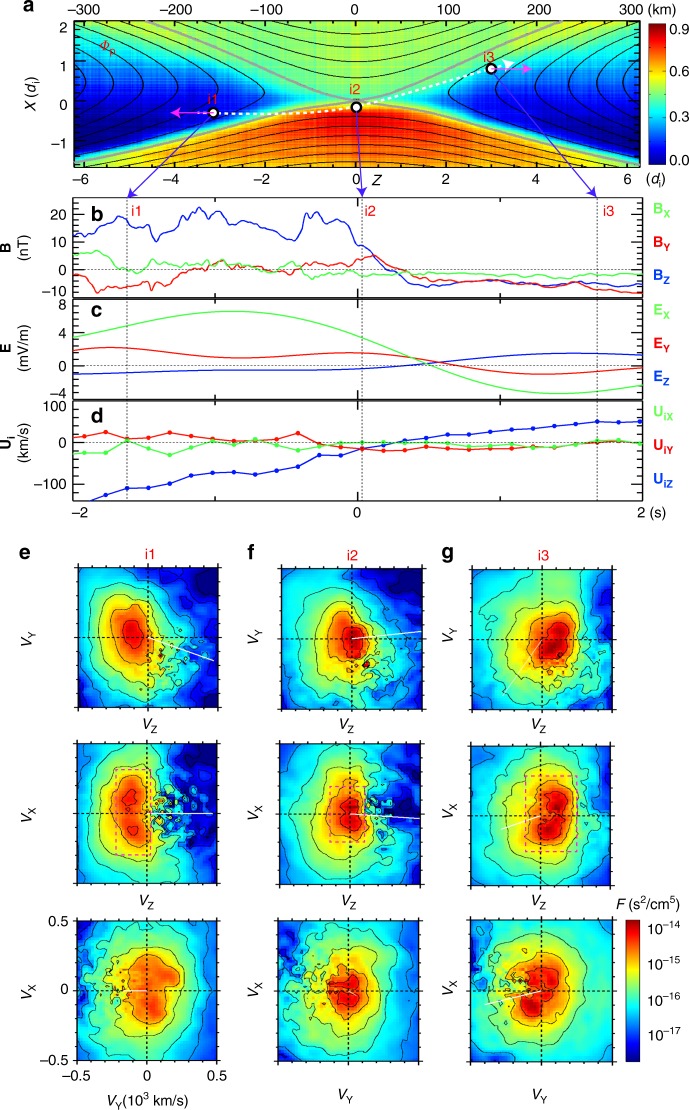
MMS measurements of ion energization during magnetopause reconnection. **a** MMS trajectory sketched on the in-plane plasma potential from a PIC simulation. The upper half (*X* > 0) is the magnetosheath (high-density side), while the lower half (*X* < 0) is the magnetosphere (low-density side). **b** The magnetic field vector components. **c** The electric field vector components (only for frequencies *f* < *f*_ci_ ~ 0.5 Hz, using the upstream magnetic field strength). Note that *E*_*Y*_ is about 1.6 mV m^−1^ near the X-line (i2). **d** The ion bulk flow velocity vector components. The direction of the in-plane ion flow vector for the time i1 and i3 is indicated by magenta arrows. **e**–**g** Ion velocity distributions measured by the MMS spacecraft (color contours represent the phase-space density) in three velocity planes for time i1, i2, and i3, which is marked with vertical dashed lines in **b**–**d**; corresponding locations marked with magenta dots in **a**. These figures indicate that ions are accelerated toward the exhaust directions in *Z* directions from X-line. Here we note that the effects of the magnetopause motion have been subtracted from both the electric field and the ion measurements

A very similar phenomenon is observed inside the ion diffusion region during the same MMS passage^11^ shown in Fig. 3. Figure 5 shows characteristics of ion acceleration in the magnetopause. Figure 5a shows the MMS trajectory in the ion diffusion region on the profile of the in-plane electrostatic potential from a PIC simulation^22^. Profiles of the magnetic field, electric field, and ion flow during the MMS crossing of the ion diffusion region are also shown in Fig. 5b–d. While the spacecraft was in the left exhaust region, a strong ion flow at the point i1 towards the −*Z* direction was observed, and an opposite ion flow to the +*Z* direction was measured at the point i3 in the right exhaust. The *V*_*Z*_-*V*_*X*_ velocity distributions (Fig. 5e–g) show counter-streaming populations (marked by magenta boxes) along *V*_*X*_ and are shifted in *V*_*Z*_, indicating that unmagnetized ions bounce around the *B*_*Z*_ reversal and are being accelerated in the *Z* direction by the in-plane electric field (gradient of the in-plane potential shown in Fig. 5a). The shift in *V*_*Z*_, same feature as the drift along *Z* shown in the ion distribution measured in MRX (Fig. 4b), is the result of acceleration by *E*_*Z*_. Hence, both MMS and MRX results support that the primary energy deposition to ions occurs due to acceleration by the in-plane electric field in asymmetric reconnection layers, similar to the case for symmetric reconnection^12–14,24^. While the observed ion acceleration in MMS is consistent with the results from MRX, the counter-streaming populations detected by MMS are not completely thermalized, an indication that ion thermalization in collisionless magnetopause reconnection requires processes occurring beyond the ion diffusion layer.

### Energy inventory in MRX and 2D simulation

In the present study, a quantitative energy inventory analysis was carried out for asymmetric reconnection layers in MRX and compared with our 2D numerical simulations. As previously done for symmetric reconnection^13,14^, the present analysis examined the inventory of the Poynting vectors, enthalpy and energy flows, and heat flux across the entire reconnection region, but without guide field. Again, more than 50 % of the incoming magnetic energy is converted to particle energy, while the electron energy gain increases, in comparison with the case of symmetric reconnection.

**Fig. 6:**
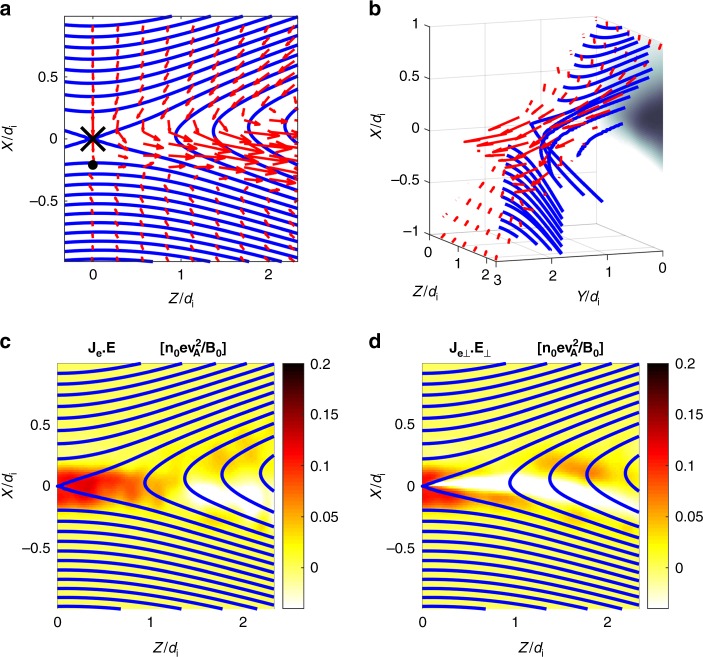
Electron dynamics calculated by our 2D numerical simulation. The presentation format is the same as Fig. 2 data from MRX. **a** Electron flow vectors are plotted together with reconnecting field lines. The X marks the X-line and the black circle denotes the stagnation point of in-plane electron flow. **b** 3D views of electron flow vectors with respect to the reconnecting field lines. Because of the dipole structure of out-of-plane magnetic field, the reconnection plane is tilted in the *Y*-axis. in the high-density (upper) side. Note that strong electron current in *Y*-direction at the stagnation point. **c** Energy deposition to electrons through **j**_e_·**E** is concentrated in the electron diffusion region around the stagnation point. **d** Perpendicular component, **j**_e⊥_∙**E**_⊥_ is much larger than *j*_e||_*E*_||_ and concentrated in the electron stagnation point, while contribution of **j**_**e||**_ ∙**E**_**||**_ is notable at the exhaust as seen in **c** and **d**. Some of the minor differences from Fig. 2 should come from the difference of boundary condition set up in the simulation

In order to clarify the energy partitioning inventory, a particle-in-cell simulation was carried out with the code VPIC^25^ for an asymmetric current sheet with no guide magnetic field. The simulation domain is *L*_Z_ × *L*_*X*_ = 4200 × 2100 cells = 40*d*_i_ × 20*d*_i_ (*d*_i_ is the ion inertial skin depth on the high-density side) for an asymmetric current sheet with no guide magnetic field. The MRX data agree remarkably well with results from our 2D simulation described in detail in Methods section. In Fig. 6, we plot our VPIC simulation data in the same format as that of the MRX data presented in Fig. 2. We observe striking similarity in the features of the flow dynamics of electrons as well as energy deposition profile while some minor differences are seen. The data are taken from a sub-domain of size ~2.5*d*_i_ × 2.5*d*_i_ around the X-line. The electron in-plane flow stagnation point in Fig. 6a is seen to be shifted to the low-density side from the X-line in agreement with the data of MRX (Figs. 1b and 2a, b). The 3D views of magnetic field lines and electron flow vectors in Fig. 6b show qualitatively similar features as in Fig. 2b. The work done on the electrons by the electric field is dominated by the perpendicular electric field, both in simulation and MRX, as shown in Fig. 6c, d and 2c, d, respectively. The ion acceleration mechanisms and ion flow velocity profiles are also verified in the present simulation as was done so before^13^. We note, however, that the parallel contributions, *j*_e||_*E*_||_, differ somewhat between the MRX data and the simulation. This is most likely caused by differences in the boundary conditions between the two, which cause differences in the parallel electron return currents that flow along the magnetic separatrices. We also note there should be some 3D effects in MRX causing some differences between the two data sets.

A quantitative energy inventory analysis was carried out for both in MRX data and in our simulation. In the MRX measurements, about 55% of the incoming magnetic energy is transferred to plasma particles at a remarkably fast speed (~0.2 V*_A_) similar to the results from symmetric reconnection^13,14^ as shown in Table 2. Regarding energy partitioning for the case of asymmetric reconnection, the agreement between MRX data and our 2D simulation is notable. While the energy partition rate does not change much with asymmetry, the overall ion energy gain decreases due to both the smaller in-plain electric field in the high-density-side of the exhaust and the smaller ion flow pattern near the low-density-side separatrices. The total ion energy gain near the low-density-side separatrices is noticeably small due to the low ion density and inflow flux, while the deposition rate to electrons increases due to extra-energy deposition at the low density-side of the exhaust region where *j*_**e||**_*E*_**||**_ is notable.

**Table 2:** Energy inventory during magnetic reconnection

	Magnetic energy inflow (1.0)	Magnetic energy outflow	Energy deposition to ions	Energy deposition to electrons	References
Symmetric	MRX data	0.45	0.35	0.20	^13, 14^
Symmetric	Simulation	0.42	0.34	0.22	^13, 14^
Asymmetric	MRX data	0.44	0.31	0.25	^10^, this study
Asymmetric	Simulation	0.43	0.32	0.25	This study

Energy partition measured in MRX for symmetric and asymmetric reconnection is compared with our 2D simulation results. Typical errors of MRX data in this table is about 10%

The length of the whole reconnection layer (2*L*) of the magnetopause was estimated to be ~250 km, which is equivalent to the total scale length of the MRX layer normalized by the ion skin depth. We have compared the energy partition in the MRX reconnection layer with the data from the recent encounter of MMS with the magnetopause. While some recent MMS measurements observed an equal amount of magnetic energy deposited to electrons and ions in the reconnection region, the limited data from MMS satellite passages in the whole reconnection region makes it difficult to determine the exact partitioning of inflowing magnetic energy at this time (no data from the flux inflow region; see Fig. 6).

Regarding the observed high rate of electron energy deposition in the present study, we should note that the measurements were carried out in the proximity of the X-line within a few ion skin depths. There exists a notable difference between the present data from both MMS and MRX and that of Phan et al.^26^, where much smaller increase of the electron temperature rise was measured at the site far from the X-line in the exhaust region (~10*d*_i_). For crossings near the X-line, such as the case shown here, the reversal of the reconnecting field and high rate of deposition at the nearby stagnation point is expected.

## Discussions

Both in laboratory and space plasmas, we have comparatively studied, in the context of two-fluid physics, the physical mechanisms responsible for the conversion of magnetic energy to plasma particle energy in a strongly asymmetric reconnection layer (*n*_1_/*n*_2_ ~ 10). Despite huge differences between the scale lengths of the reconnection layers (2*L* ~ 30 cm in MRX vs 250 km in the magnetopause) and the ion skin depths (*d*_i_ ~ 5–6 cm in MRX, vs ~ 50 km in the magnetopause), remarkably similar characteristics are observed regarding the dynamics of electrons and ions, as well as energy deposition profiles and energy partitioning. In addition to the earlier observational verification of electrons’ motion frozen to field lines outside of the electron diffusion region both in MRX^12–14^ and MMS^11^, an excellent agreement was found between the dynamics and energetics of electrons (Figs. 1 and 2 vs. Fig. 3) and ions (Fig. 4 vs. Fig. 5). This agreement demonstrates that the same two-fluid mechanisms in 2D analysis operate well in both systems despite vastly different scales (~10^6^), while various 3D phenomena including micro-fluctuations are expected to be involved^22^.

In the reconnection layers of MRX and at the magnetopause with negligible guide field (*B*_G_ < 0.1*B*_rec_; *B*_rec_ is the strength of the reconnecting field component), it was found that the energy deposition to electrons primarily occurs through **j**_e⊥_·**E**_⊥_ near the electron diffusion region with some contribution in the low-density side of the exhaust. Furthermore, it was found in MRX and numerical simulation study that a sizable amount (55%) of inflow magnetic energy is converted to ions (~30%) and electrons (~25%) in an asymmetric reconnection layer of a few ion skin depths. While this measurement is yet to be verified in the magnetosphere measurement by MMS, future MMS inventory study by multiple MMS crossings would be able to address this key result.

The reconnection rate is measured in MRX based on 2D analysis by monitoring an average velocity of magnetic field lines (or following flux lines) in the reconnection plane with a size of a few ion skin depth. The observed values agree well with the measured values of local reconnection electric field measured both in MRX and MMS averaged over an ion gyro time (*ω*_ci_^−1^) and ion skin depth (~*d*_i_), and the results translate to a reconnection speed of 0.2–0.3 V*_A_. The fast reconnection rate can be explained by the Hall effects in the ion diffusion region^1–3^. However, the Hall term alone does not create the energy dissipation necessary for conversion of magnetic energy^2^. It has been considered that the electron pressure tensor term and/or fluctuations can generate energy dissipation particularly at the electron diffusion region^2,27–29^. Wave activities near the diffusion region^30,31^ may contribute to fast reconnection as well as to fast energy conversion to electrons, although the present comparative study could not find a conclusive relationship between the wave amplitude and the reconnection rate.

Recently, a concept of a super-cluster cubesat system has been developed, which is based on a 2D (11 × 11) or 3D (5 × 5 × 5) satellite grid with an equal distance in Earth’s magnetosphere. Since the key two-fluid physics occurs in the scale length of 1–200 km, optimal distance between adjacent satellites is 2–50 km, such that the grid size can be 20–500 km. This system can be used for measuring the structure of the magnetic reconnection layer at a given time, directly contributing to understanding the global dynamics of magnetic reconnection in space. It can also provide data of the energy inventory in space, as well as data of the relation between fast fluctuations in the electron diffusion region and the reconnection rate.

## Methods

### Asymmetric reconnection in MRX

In MRX, a density asymmetry is generated during the plasma formation period due to the inductive electric field, *E*_TF_, from the increasing TF coil current^9,10^. For this experimental campaign, the direction of E_TF_ during the plasma formation is radially outward between the flux cores, as illustrated in Fig. 1a. In this configuration, ions are transported radially outward along *E*_TF_, generating a radial density asymmetry. The density asymmetry during the pull reconnection period depends on the TF current waveform, the gas species, and the fill pressure. A plasma with more massive ions has a larger density asymmetry during the quasi-steady period due to an inertia effect. In the present experiment, we use helium to create an asymmetric plasma. The helium fill pressure is varied for further control of the density asymmetry ratio up to 10.

### Numerical simulations

**Fig. 7:**
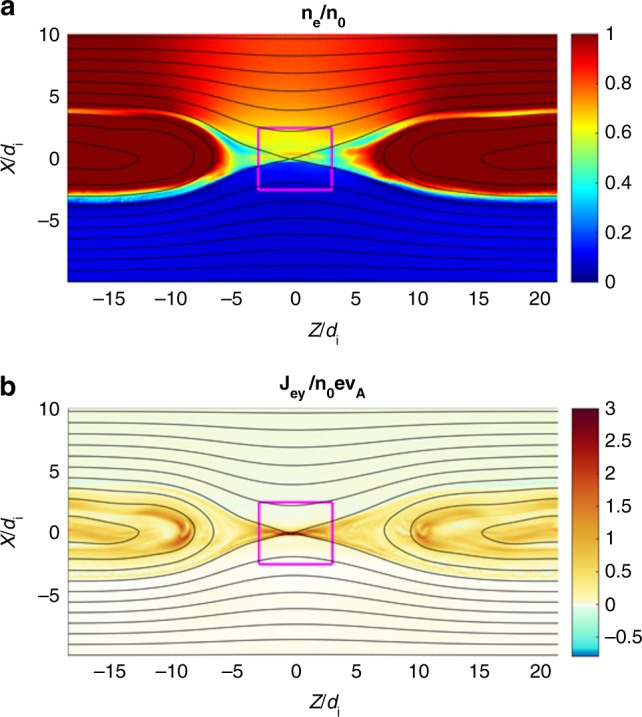
Overview of VPIC simulations. The **a** density profile and **b** out-of-plane electron current from the VPIC simulation of asymmetric reconnection are shown. The energy budget was evaluated over the magenta box centered on the X-line

In order to compare the MRX data with a numerical calculation, a particle-in-cell simulation was performed with the code VPIC^25^ of an asymmetric current sheet with no guide magnetic field. The simulation domain is *L*_*Z*_ × *L*_*X*_ = 4200 × 2100 cells = 40*d*_i_ × 20*d*_i_ (*d*_i_ is the ion inertial skin depth on the high-density side). The initial reconnecting field is *B*_z_ = 1/2[(*B*_2_ – *B*_1_) + (*B*_2_ + *B*_1_) tanh(*X*/*L*)], where *B*_2_ refers to the low-density side, *B*_1_ refers to the high-density side, and the scale length *L* *=* 1*d*_i_. The electron and ion temperatures are initially uniform and equal, and the density profile, which jumps by a factor of 10 across the current sheet, is selected to ensure hydrodynamic pressure balance. These parameters were selected to mimic conditions in the MRX experimental device. The boundary conditions are periodic in *X*, and conducting for fields and reflecting for particles in *Z*. Other numerical parameters include a reduced electron-to-ion mass ration of *m*_i_/*m*_e_ = 100 and the electron plasma-to-cyclotron frequency ratio of *ω*_pe_/*ω*_ce_ = 2. The numerical particles are given different weights on each side of the current sheet so that there are 400 particles per cell per species on each side (giving 7 billion total). Additional details of the asymmetric current sheet set-up are found in ref. ^29^. The energy balance was computed over a box of size *L*_*X*_ × *L*_*Z*_ = 2.5*d*_i_ × 3*d*_i_ centered on the X-line at time *t* = 96/*ω*_ci_, when reconnection was quasi-steady, as shown in Fig. 7. The VPIC code is a general-purpose PIC simulation and available online (https://github.com/lanl/vpic).

**Fig. 8:**
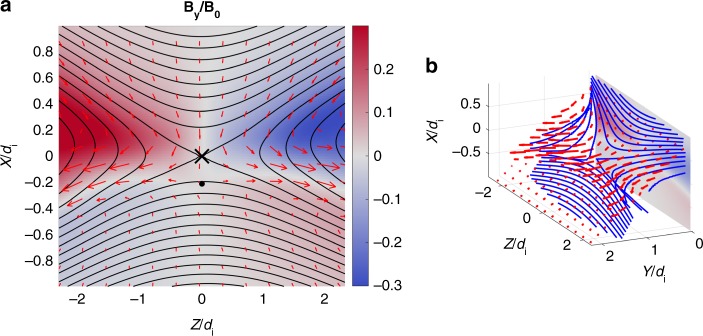
Electron dynamics in the VPIC simulation. **a** The out-of-plane Hall magnetic field is shown with sample in-plane field lines electron flow vectors. The X marks the X-line and the dot marks the in-plane electron flow stagnation point, respectively. **b** A 3D representation of the magnetic fields lines and electron flow vectors demonstrates similar electron dynamics presented in Figs. 1, 2

To aid in comparison to the MRX experimental data, Fig. 8a shows the out-of-plane Hall magnetic field, sample magnetic field lines, and the electron flow plotted in a similar manner to Fig. 1b from the main text. The bipolar Hall field structure is very similar to the experimental data. Figure 8b shows a 3D view of the data to show the tilt of the plane of the field lines near the X-line produced by the Hall field.

## Electronic supplementary material

Description of Additional Supplementary Files

Supplementary Movie 1

## Data Availability

MMS data for the event on 16 October 2015 are available at the MMS Science Data Center (https://lasp.colorado.edu/mms/sdc/public/search/) by specifying the date and time (13:05:00–13:08:00). MRX and simulation data can be found in the Data Space of Princeton University (http://arks.princeton.edu/ark:/88435/dsp01x920g025r).
