# Supplementary material for: The two-fluid dynamics and energetics of the asymmetric magnetic reconnection in laboratory and space plasmas
Source: Nat Commun. 2018 Dec 6;9:5223. doi: 10.1038/s41467-018-07680-2 (PMC6283883; doi:10.1038/s41467-018-07680-2)
Supplement: Supplementary file 1 — Description of Additional Supplementary Files [file 41467_2018_7680_MOESM1_ESM.pdf]

Description of Additional Supplementary Files

File Name: Supplementary Movie 1
Description: **Movement of the field lines during asymmetric reconnection in MRX.** Color contours on the *X*-*Z* plane show the profile of the out-of-plane magnetic field in Fig. 1(b). The blue lines represent the field lines measured by a 2D magnetic probe array. The field lines move from both the high-density (upper) side and low-density (lower) side toward the center of the reconnection layer (X-line) where field lines break, reconnect, move away from the X-line. Due to the asymmetry in magnetic field profile, the plane where the field lines move on the high-density side is tilted significantly with respect to the *Y* axis.
